# Environmental Gradients in Lizard Colouration

**DOI:** 10.1002/ece3.71012

**Published:** 2025-02-28

**Authors:** Lekshmi B. Sreelatha, Pedro Tarroso, Ossi Nokelainen, Zbyszek Boratyński, Miguel Angel Carretero

**Affiliations:** ^1^ CIBIO‐InBIO Associate Laboratory, Research Centre in Biodiversity and Genetic Resources University of Porto Vairão Portugal; ^2^ Departamento de Biologia Faculdade de Ciências da Universidade do Porto Porto Portugal; ^3^ BIOPOLIS Program in Genomics, Biodiversity and Land Planning, CIBIO Vairão Portugal; ^4^ Open Science Centre University of Jyväskylä Jyväskylä Finland; ^5^ Department of Biological and Environmental Science University of Jyväskylä Jyväskylä Finland

**Keywords:** brightness, dorsal colouration, ecogeographic rules, lizards, melanisation, thermal melanism

## Abstract

Environmental pressures shape animal colouration, facilitating adaptation to local conditions. However, the extent to which climatic gradients drive colour variation in a species across its distributional range remains unclear. Here, we tested whether the dorsal colouration of Lusitanian wall lizards (*Podarcis lusitanicus*) varies spatially in response to environmental gradients across its distribution in the north‐western Iberian Peninsula. We estimated dorsal colour brightness (i.e., lightness) from multispectral photographs of 463 animals, originating from 21 locations distributed across the species range. We studied direct and indirect (mediated by body mass) relationships between environmental variables and the lightness of lizards, by piecewise structural equation modelling. We simultaneously tested predictions from Gloger's (darker colouration in warm and humid environments), thermal melanism (darker colouration in colder environments), photoprotection (darker colouration in areas with higher intensity of solar radiation) and Bergmann's (larger body size in colder environments) hypotheses. We found that the lightness of lizards best follows predictions of Gloger's hypothesis for humidity, but not supporting the photoprotection hypothesis, independent of the populations' shared ancestry and geographic location. We found no support for direct thermal melanism, as temperature was not directly associated with lightness. Instead, the indirect effect of temperature on lightness through body size was detected. Consistent with Bergmann's hypothesis, lizards in colder regions tended to be larger and darker. Our study indicates that the evolution of lizard dorsal colouration is driven by variable climatic factors. Experimental tests are necessary to assess the mechanisms driving climatic effects on colouration across diverse environments, advancing beyond the simplistic correlations suggested by ecogeographic hypotheses.

## Graphical abstract

In our study, we examined how environmental gradients influence dorsal colour variation in Lusitanian wall lizards across their range in the Iberian Peninsula. We found that lizard dorsal lightness is primarily associated with humidity, supporting Gloger's hypothesis, while body size influences dorsal lightness indirectly through Bergmann's hypothesis, with lizards being larger and darker in colder regions. Further experimental research is needed to clarify the mechanisms behind these climatic effects on colouration.

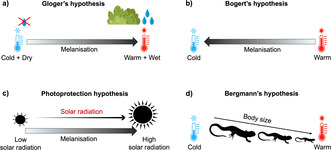

## 1 Introduction

Understanding the maintenance of diversity in animal colouration, both within and among species, is a key issue in evolutionary ecology (Cuthill et al. 2017; Endler and Mappes 2017). Animal colouration serves various functions, including camouflage, thermoregulation, defence, and communication (Gamble et al. 2003; Merilaita et al. 2017; Smith et al. 2016; Stuart‐Fox and Moussalli 2009). Different environments can lead to variations in colouration among individuals and populations, influenced by both biotic (e.g., visual system of the observers, diet) and abiotic (e.g., habitat background, light conditions, thermal environment) factors, in addition to stochastic neutral processes such as genetic drift (Endler and Thery 1996; Friedman and Remeš 2017; McNaught and Owens 2002; Merilaita 2003; Nokelainen et al. 2020). Thus, colouration may be driven by a wide range of selective pressures forcing animals to adapt to their local environment (Endler 1990).

The complex interplay between coloration and diverse selection pressures acting upon it highlights its multifaceted functional significance, extending well beyond its role in visual aesthetics. All else being equal, darker colours (low reflectance) tend to absorb more heat, while lighter colours (high reflectance) reflect it more (Stuart‐Fox et al. 2017). Thus, animal coloration can have a crucial role in heat exchange, alongside other behavioural thermoregulation strategies, such as microhabitat selection, adopting certain body postures and orientation, movement between sun and shade, and adjusting activity windows (Angilletta 2009; Angilletta et al. 2010; Clusella‐Trullas et al. 2007). The variation in such vital biological responses is largely determined by the climatic gradients of their habitats as well (Mader et al. 2022; Smith et al. 2016). In consequence, selection pressure on colouration is expected to be complex and spatially heterogeneous, varying due to local habitat and climate diversity (Endler 1977). So, adaptive variation in colouration emerges as a delicate balance between multiple competing functions of colouration (such as camouflage, signalling and thermoregulation), with each of them subjected to often conflicting selective forces (Cuthill et al. 2017; Endler and Mappes 2017). These dynamics contributed to the observed patterns underlying ecogeographic hypotheses, which describe the covariation of phenotypic traits with environmental variables, including geographic position (e.g., latitude and elevation) and climate (e.g., temperature and precipitation) (Delhey et al. 2019; Gaston et al. 2008).

**FIGURE 1:**
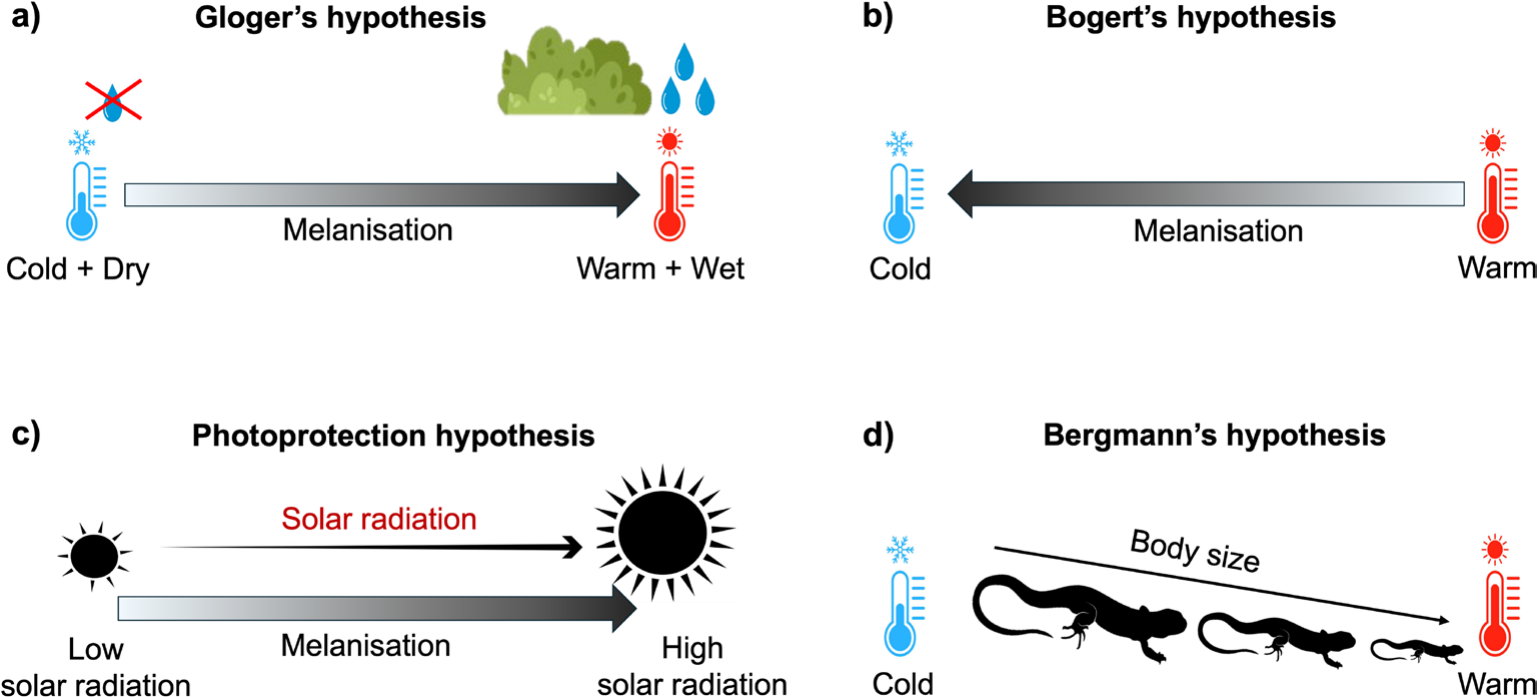
Ecogeographical hypotheses affecting the lightness and body size of animals. (a) Gloger's hypothesis (animals are darker in warm and humid areas), (b) Thermal melanism hypothesis (Bogert's hypothesis, animals are darker in colder areas), (c) Photoprotection hypothesis (animals are darker in areas with high solar incidence as a protection against UVB radiation), (d) Bergmann's hypothesis (animals have bigger body size in colder areas).

Two main hypotheses explaining ecogeographic variation in animal colouration have been raised: Gloger's hypothesis (Delhey 2017; Rensch 1938) and thermal melanism or Bogert's hypothesis (Bogert 1949, Clusella‐Trullas et al. 2007). Both primarily address achromatic variation, which ranges from light to dark (i.e., higher to lower reflectance), typically associated with the deposition of melanin pigments (McGraw 2006). Inspired by the work of C.W.L. Gloger, who recognized that birds and mammals tend to be more intensively pigmented in tropical regions (Gloger 1833), Gloger's hypothesis predicts, in its modern interpretation, that animals should be darker in warm and humid areas (Figure 1a; Delhey 2017; Rensch 1938). Originally defined for endotherms, this hypothesis accounts for intraspecific variations in colouration, but similar trends have also been observed across different taxonomic groups, also in ectotherms (Delhey 2017). Thermal melanism hypothesis predicts that at the same level of solar radiation, ectotherms living in colder regions should be darker (Bogert 1949; Clusella‐Trullas et al. 2007; Figure 1b). This adaptation may allow them to have a faster heat gain, enabling them to reach optimal operating temperatures more quickly than their lighter‐coloured counterparts (Bogert 1949; Clusella‐Trullas et al. 2007; Delhey 2018). For example, in the western Cape of South Africa, cordylid lizards exhibit a distribution pattern where melanistic species are found in cold, foggy peninsular and mountainous regions while non‐melanistic *Cordylus* spp. occupies a broader range, including coastal and typically warmer inland areas (Clusella‐Trullas et al. 2009). Gloger's rule associates darker pigmentation with warmer, humid regions, while the thermal melanism hypothesis links it to colder climates for thermoregulation. These conflicting predictions create ambiguity in regions with, for example, high humidity and low temperatures, with one hypothesis predicting darker pigmentation and the other favouring lighter pigmentation for the same location. Beside Gloger's and Bogert's hypotheses, which focus on climatic factors, the photoprotection hypothesis suggests that the degree of melanisation is linked to the protective function of melanin (Figure 1c; Law et al. 2020; Lopez et al. 2021). This pigment can act as a protective barrier against intense solar radiation, particularly the damaging impact of UV‐B rays on DNA (Mosse and Lyakh 1994; Wang et al. 2008).

The thermal melanism and photoprotective hypotheses are supported by clear adaptive mechanisms, whereas the mechanistic basis behind the predictions of Gloger's hypothesis is still unclear (Clusella‐Trullas et al. 2007; Delhey 2019; Law et al. 2020). Although this hypothesis is usually interpreted in relation with humidity, some studies suggest a number of proxies, such as latitude, vegetation structure and solar radiation, or their correlates (Delhey 2019). The diversity of functions proposed to explain the observed patterns encompass camouflage, defence against pathogens and other pleiotropic effects of melanin (Delhey 2017, 2019). Indeed, areas with warm, humid climate typically harbour denser vegetation and soils richer in organic matter, creating darker backgrounds. In these conditions, selection should favour darker colouration in animals to have effective matching with their darker backgrounds (Friedman and Remeš 2017). However, increased melanin deposition leading to darker colouration in animals may also be driven by interactions with parasites or pathogens. In humid or densely vegetated environments, where animals may face higher pathogen prevalence (Horrocks et al. 2015), increased melanin deposition may enhance immune competence through its pleiotropic effects against environmental pathogens (Delhey 2019). Pleiotropic effects of melanin production pathways make darker animals express more aggressive behaviours and stress tolerance, and better anti‐inflammatory, antipyretic and anti‐oxidative responses, as well as increased levels of sexual activity and better energy balance (Ducrest et al. 2008; Raia et al. 2010). Therefore, although the multiple plausible functions make the general applicability of Gloger's hypothesis complicated, understanding colour‐related biogeographic patterns provides a foundation to understand spatial variation in physiological and behavioural traits.

In ectotherms, thermoregulation solely depends on the heat gathered from external sources. As such, they must efficiently manage heat exchange with their surroundings to enhance their overall performance (Angilletta 2009). While the thermal melanism hypothesis suggests that melanisation enhances heat absorption, offering a thermoregulatory advantage in colder climates, it is important to acknowledge that there is often a suite of multiple traits contributing to the thermal benefits of colouration in ectotherms (Stuart‐Fox et al. 2017). Namely, the rate and extent of radiant heat absorption are also influenced by body size, along with behavioural thermoregulation (Clusella‐Trullas et al. 2007). Under the same environmental conditions, heat gain is faster in smaller ectotherms than in the larger ones. Larger ectotherms have greater thermal inertia allowing better heat retention once warmed, but at the expense of slower heat gain (Carothers et al. 1997; Clusella‐Trullas et al. 2007; Zamora‐Camacho et al. 2014). Despite needing more time to reach equilibration, larger animals can achieve higher equilibrium temperatures compared to smaller ones (Clusella‐Trullas et al. 2007). Originally based on endotherms (for ectotherms see Angilletta et al. 2004), Bergmann's ecogeographic hypothesis predicts that animals are larger in colder areas (Figure 1d; Bergmann 1847). Since darker animals are expected to heat faster and larger animals to display greater thermal inertia, a combination of darker coloration and larger body size is expected to enhance heat gain and heat retention under cold conditions, although this relationship is heavily influenced by the specific microhabitat conditions and behaviour of the animals (Stuart‐Fox et al. 2017). In warm climates, larger ectotherms may need to be brighter to decrease heat loads and avoid overheating (Clusella‐Trullas et al. 2007, 2008). As such, melanisation and body size have to be optimised to suit different thermal environments across a species range. However, the relationship between these traits can be further complicated by various selective forces, including life history (such as growth, maturation, survival, and reproduction in different thermal environments), predation pressure, activity time, as well as affected by stochastic processes related to genetic drift (Angilletta et al. 2004; Clusella‐Trullas et al. 2007, 2009; González‐Morales et al. 2021). In other words, generalisations about relationships between temperature and body size in ectotherms may not be simple, and great caution is advised before attributing variations in skin colour and body size exclusively to thermoregulatory advantages (Angilletta and Dunham 2003; Stuart‐Fox et al. 2017).

In this study, we investigate the role of environmental variables in the variation of achromatic dorsal colouration (lightness hereafter) using the Lusitanian wall lizard, *Podarcis lusitanicus* Geniez, Sá‐Sousa, Guillaume, Cluchier & Crochet, 2014, as the model organism. This lizard species is distributed widely across different environments in the north‐western Iberian Peninsula, from sea level to elevations reaching 1850 m (m.a.s.l.) and demonstrates broad variation in dorsal patterns. Its distribution across diverse environmental conditions provides a unique opportunity to test the generality and potential contradictions between ecogeographic hypotheses. To investigate the ecogeographic patterns of lightness variation in our model species, we collected individual lightness data in a number of populations representing the environmental variability experienced by the species. Using these data, we applied structural equation modelling to simultaneously test various ecogeographic hypotheses such as: (1) lizards are darker in warmer and more humid areas in line with Gloger's hypothesis (Delhey 2019); (2) they are darker in colder areas, consistent with the thermal melanism hypothesis (Clusella‐Trullas et al. 2007, 2009); (3) they are darker in areas with intense solar radiation consistent with the photoprotection hypothesis (Law et al. 2020); and (4) they are darker and larger in colder regions, in line with Bergmann's hypothesis (Bergmann 1847). We accounted for sexual dimorphism in body size and colouration, as well as potential geographic variation in confounding environmental factors, including various climatic variables, elevation and habitat.

## 2 Materials and Methods

### 2.1 Study Species and Sampling

The Lusitanian wall lizard, *P. lusitanicus* Geniez, Sá‐Sousa, Guillaume, Cluchier & Crochet, 2014, which was recently elevated to species rank (Caeiro‐Dias et al. 2021) is a small (~1.5–5.0 g) lacertid lizard, endemic to the north‐western Iberian Peninsula. Its distribution encompasses both Mediterranean and Atlantic areas (Carretero et al. 2022; Sillero et al. 2009), and it underwent in situ microrefugia dynamics during the Pleistocene (Rato et al. 2025). This is a diurnal and highly saxicolous species using rocks (generally granite or schist) for thermoregulation, shelter, and a food source more frequently than other species of the genus (Gomes et al. 2016). *P. lusitanicus* inhabits diverse environmental conditions and exhibits substantial variation in dorsal pattern, making it an ideal candidate for our study. With its distribution restricted to the north‐western Iberian Peninsula, we were able to sample nearly its entire range, collecting 470 adult lizards from 21 sampling locations (populations hereafter, Table S1; Figure S1), which represent most of the variation in climatic (temperature, precipitation, solar radiation) and topographic (elevation) features within the species' range. Lizards were captured during the reproductive season (Carretero et al. 2022), between April and July of 2022 (18 populations in Portugal) and 2023 (three populations from Galicia, Spain). We measured the snout‐to‐vent length (SVL; to the nearest 0.01 mm with an electronic calliper, mean + SE = 51.55 ± 0.30 mm) and body mass (to the nearest 0.01 g with a portable digital scale, 2.66 ± 0.05 g), and collected multispectral photographs (see below) before releasing them back within the same day of capturing. Out of the initial 470 animals, seven were excluded due to blurred or out‐of‐focus photographs, leaving 463 animals for further analysis.

### 2.2 Photography and Image Analysis

To quantify the reflectance in both visible and UV spectrum, we took photographs of the captured lizards using a customised full spectrum Samsung NX1000 camera equipped with minimum light absorption Novoflex Noflexar 35 mm lens. For the human‐visible spectrum, we used a UV–Infrared (IR) blocking filter (Baader CMOS UV/IR‐Cut bandpass filter) in front of the lens, which only transmits wavelengths from 420 to 685 nm. For the images in the ultraviolet spectral range, a UV pass filter (Baader U filter) was used, which transmits wavelengths from 320 to 380 nm. To standardise the photographs for ambient light conditions, a grey reflectance standard (Zenith Polymer Standard, SphereOptics, Herrsching Germany) reflecting light equally at 50% between 300 and 750 nm was included in the photographic scene. A photographic umbrella was used to minimise the glare, and a scale bar was placed in all photographs for standardising and scaling the images uniformly. We took photographs of the most common backgrounds (such as granite walls, rocks etc) used by the lizards as their visual backgrounds in their habitat. Each of the backgrounds photographed were at least 10 m apart from each other, ensuring the variability of the backgrounds. All images were taken at the same distance with the same camera settings (camera's settings were kept constant among the photographs; aperture priority and with constant ISO was used) in the field using the natural ambient light and saved in RAW format.

All image analyses were carried out using ImageJ (Abràmoff et al. 2004). Multispectral images were created using the ‘multispectral image calibration and analysis toolbox’ in Image J (Troscianko and Stevens 2015). Images from visible and UV spectrum were aligned and the 50% reflectance standard was selected in order to normalise the images standardising the radiance and light conditions. All images were rescaled according to a 30 mm scale bar (Stevens et al. 2007). Dorsal part of each lizard, excluding the tail, head and limbs, was selected as the region of interest. The generated multispectral images were composed of bandpass layers corresponding to the long‐wavelength (LW, red), medium‐wavelength (MW, green), short‐wavelength (SW, blue) and ultraviolet (UV, ultraviolet) parts of the spectrum. We calculated the brightness of the dorsum (lightness hereafter) and the visual backgrounds (habitat lightness hereafter) as (LW + MW + SW + UV)/4, as a measure of how dark or bright they are across the entire spectrum (Stevens et al. 2014). Dorsal colour lightness was calculated from raw camera responses without making any assumptions about the receiver vision.

### 2.3 Climatic and Topographic Variables

We collected information about the environmental variables potentially influencing lizard activity, including their seasonality, which were selected and downloaded from different sources. We extracted eight climatic variables at a 30 arcsec spatial (1 km) resolution for all the sampling locations from Chelsa (version 2.1), which provides spatially interpolated monthly averages of weather station data from the period between 1981 and 2010, along with 19 bioclimatic variables that further summarise climate patterns (D. Karger et al. 2018; D. N. Karger et al. 2017). From Chelsa, we downloaded variables describing sampling locations, such as annual mean temperature (hereafter mean ambient temperature, °C), temperature seasonality (T_season_, °C/100), max temperature of warmest month (T_max_, °C), temperature annual range (T_annual range_, °C), annual precipitation (P_annual_, kg m^−2^ year^−1^), precipitation seasonality (P_season_, kg m^−2^), precipitation of warmest quarter (P_wq_, kg m^−2^ month^−1^) and humidity level (aridity index, ratio of mean annual precipitation to mean annual evapotranspiration; higher index represents more humid areas). We extracted solar radiation data (MJ m^−2^ day^−1^) from Worldclim 2.0 (Fick and Hijmans 2017) at a 30 arcsec spatial (1 km) resolution for all the sampling locations for the duration 1970–2000. Solar radiation is calculated by interpolating weather station data with the covariates of cloud cover, distance from the oceanic coast, elevation, and top atmosphere incident radiation calculated from latitude as covariates in model building (Fick and Hijmans 2017). We extracted elevation data (30 m resolution) from the Shuttle Radar Topography Mission (SRTM; data available at http://srtm.csi.cgiar.org/) and resampled it by bilinear method to match with the extent and resolution (1 km) of other variables. Vegetation index, a measure of the photosynthetic activity of plants, has been included as a proxy of the vegetation cover (Estrada‐Peña et al. 2014). For that, we obtained the normalised difference vegetation index (NDVI, hereafter vegetation cover) data from the MODIS Terra satellite MOD13Q1 product, with 250 m spatial resolution and 16 days temporal resolution for the period between 2002 and 2022. The time series were summarised using harmonic regression to capture the seasonal pattern in each pixel, resulting in 11 coefficients that represent different harmonic frequencies in the time series (Estrada‐Peña et al. 2014). Final datasets were spatially averaged to 1 km resolution. The coefficient representing the largest time window was used in the further analysis.

### 2.4 Statistical Analysis

To test relationships between the environmental variables and lightness of animals, we applied piecewise structural equation modelling (pSEM; R package piecewiseSEM v2.3.0; Lefcheck 2016) in combination with linear mixed models (R package lme4 v. 1.1–35.3; Bates et al. 2015). To reduce the skewness in the data and to homogenise the variance, lightness and body size data were log_10_ transformed. All continuous variables were centred and scaled prior to the analyses. Structural equation modelling tests direct and indirect relationships (paths) between multiple dependent variables and predictors in complex systems (Grace et al. 2010; Lefcheck 2016). The method applies Shipley's test of d‐separation to test for missing paths between unconnected variables to ensure that there are no missing relationships, improving fit of the analysis (Lefcheck 2016; Shipley 2000). Overall model fit was verified with Fisher's C statistic and Akaike's Information Criterion corrected for small sample size (AICc; Badiane et al. 2022, Lefcheck 2016).

**FIGURE 2:**
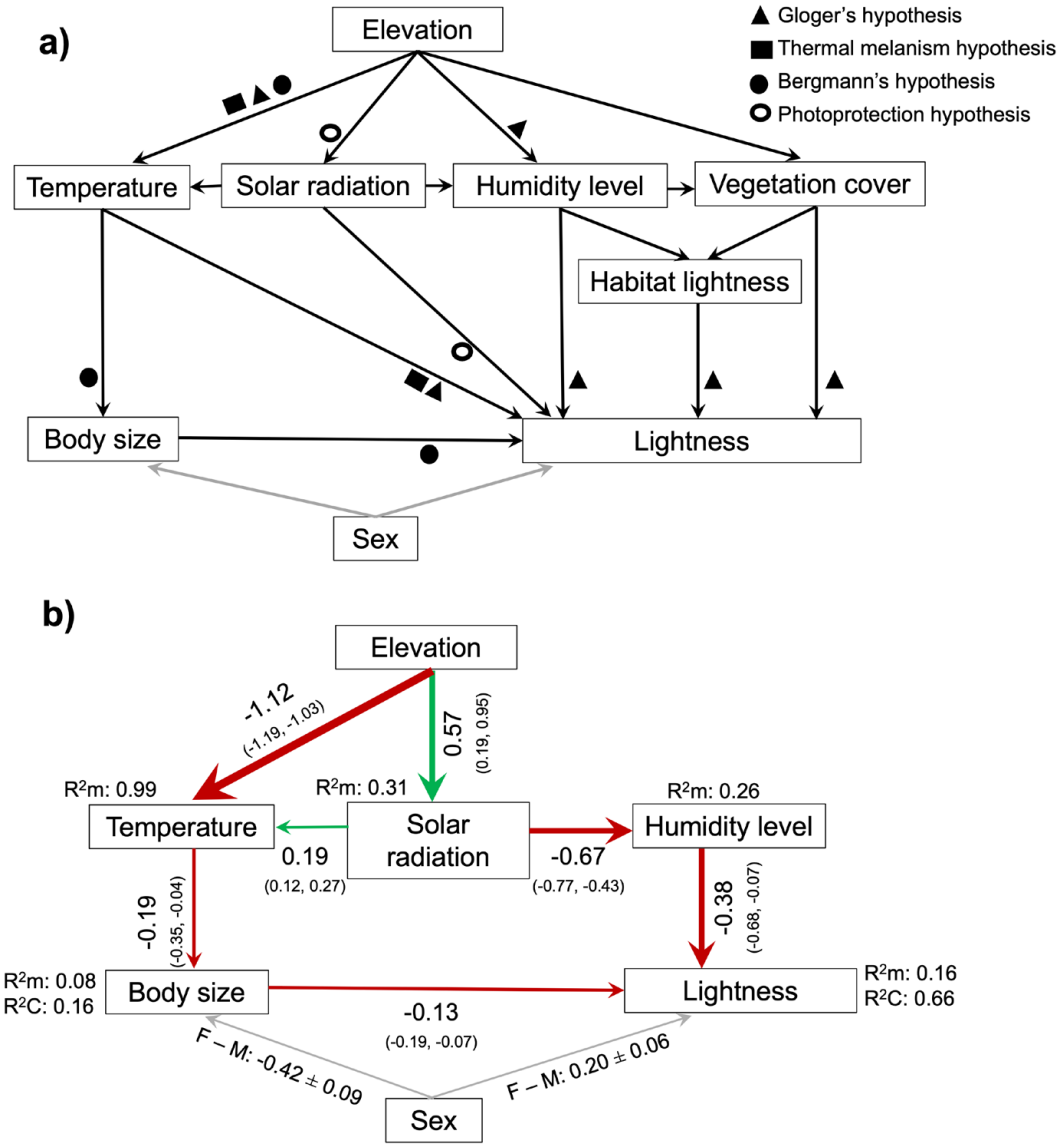
(a) A priori model showing the predicted effects of environmental variables on the lightness of *Podarcis lusitanicus* lizards (*N* = 463) across the sampled populations (*N* = 21). Each single‐headed arrow represents a direct causal path (e.g., temperature affects lightness directly). Effects can be indirect as well (e.g., temperature affects body size and therefore lightness). Geometric shapes near each path represent the ecogeographic hypothesis relevant for that path. Grey arrows represent the possible effect of sex as a categorical factor on body size and dorsal colour brightness. (b) Best selected path diagram from piecewise SEM showing the direct and indirect paths between lightness, body size (measured by snout‐vent length) of *P. lusitanicus* lizards (*N* = 463) and environmental variables across the sampled populations (*N* = 21). Each single‐headed arrow represents a statistically significant direct causal path and arrow thickness is proportional to their effect size. The numbers near the arrows show standardised path coefficients (with 95% CI) indicating the magnitude of the effect of one variable upon another. Red and green arrows represent the negative and positive relations, respectively. Numbers near sex effect report the contrast between estimated marginal means of body size and brightness for female and male (F‐M).

The initial pSEM analysis was built on the predictions from the ecogeographic hypotheses (Figure 2a). It is predicted that the lightness of animals is directly affected by variation in humidity level (Gloger's hypothesis), vegetation cover (Gloger's hypothesis), ambient temperature (Gloger's and thermal melanism hypotheses), solar radiation intensity (Photoprotection hypothesis), habitat lightness (Gloger's hypothesis) and body size of animals (Figure 1). A separate path testing the direct effect of ambient temperature on body size (Bergmann's hypothesis) was included as well (Figure 2a). Being more robust and occupying exposed areas during the breeding season, males may exhibit distinct colours compared to females, either to camouflage in open environments or to signal their quality to rivals and potential mates (Gomes et al. 2016; Sillero and Gonçalves‐Seco 2014). So, we controlled for sexual dimorphism by adding sex as a factor in the analyses (presenting marginal means for each level given the model structure; Table 1). To confirm the significance of sex as a categorical variable, we performed post hoc analyses using pairwise comparisons of estimated marginal means between males and females (R package emmeans v.1.10.2; Lenth 2024). The paths missing from the initial pSEM model (e.g., direct effect of mean ambient temperature on humidity level) were added to the analysis, as suggested by the d‐separation test. The complete model included the assumption of covariation between elevation and mean ambient temperature (*r* = −0.97, df = 19, *p* < 0.001) to account for the unmeasured sources of variance that are influencing the relationship between these two variables (Lefcheck 2016). We applied stepwise backward elimination to reduce the complexity of the final model (Figure 2b; Table S2) and calculated standardised coefficients of pSEM effects, and their 95% confidence intervals (R package semEff v. 0.6.1; Murphy 2022). The following models were fitted simultaneously in the final model: (1) lightness ~ humidity level + mean ambient temperature + body size + sex; (2) body size ~ mean ambient temperature + sex; (3) solar radiation ~ elevation; (4) mean ambient temperature ~ elevation + solar radiation + humidity level, (5) humidity level ~ elevation + solar radiation, where models 1 and 2 were fitted with linear mixed models with population as a random factor and models 3–5 were fitted with general linear models.

**TABLE 1:** Summary of the best fit piecewise SEM model (Fisher's C = 9.47, df = 16, *p* = 0.893) showing the path between lightness of *Podarcis lusitanicus* lizards (*N* = 463) and environmental variables across the sampled populations (*N* = 21).

Response	Predictors	*β*	*S.E*	*d.f*	*p*
Lightness	Humidity level	−0.38	0.16	18.35	0.029
	Mean ambient temp.	−0.06	0.17	18.15	0.733
	Body size	−0.13	0.03	443.38	< 0.001
	Sex	—	—	1.00	< 0.001
	Male	−0.05	0.16	18.78	0.774
	Female	0.16	0.17	19.54	0.355
Humidity level	Elevation	0.48	0.27	18.00	0.096
	Solar radiation	−0.67	0.27	18.00	0.022
Solar radiation	Elevation	0.57	0.19	19.00	0.009
Mean ambient temp.	Elevation	−1.12	0.04	17.00	< 0.001
	Solar radiation	0.20	0.04	17.00	< 0.001
	Humidity level	−0.05	0.03	17.00	0.107
Body size	Mean ambient temp.	−0.20	0.08	18.68	0.023
	Sex	—	—	1.00	< 0.001
	Female	−0.26	0.09	35.94	0.008
	Male	0.16	0.09	27.96	0.066

*Note:* All continuous predictors were standardised (centred and scaled) prior to the analysis. Lightness and body size were log10 transformed prior to standardisation and fitted into linear mixed models with population as random factor using the package lme4 (R package lme4 v. 1.1–35.3; Bates et al. 2015). All the other relations were fitted into general linear models using the built in glm() function in R. Sex is included as a categorical factor. The complete model included assumption of covariation between elevation and mean ambient temperature, to account for the unmeasured sources of variance between these two variables (see Table S2 for additional details). (*β* = standardised estimate, S.E = standard error, d.f = degrees of freedom. Mean ambient temp. stands for mean ambient temperature).

In a separate analysis, in addition to the effect of individual climatic variables, we also evaluated the effect of the overall environment on the lightness of animals. For this, we identified the major axes of environmental variation through a Principal Component Analysis (PCA, R package psych v. 2.4.3; Revelle 2024) of the selected 10 environmental variables (Table S3). Principal components (PCs), each explaining > 5% of the variance in the data with eigenvalues > 1, were selected for further analyses (Table S3). To explore the relations between the climatic PCs and the lightness of the lizards, we conducted a linear mixed model analysis for the selected PCs as predictor variables and the lightness of the lizards as the response variable (R package lme4 v. 1.1–35.3; Bates et al. 2015). Sex and body size were added as cofactor and covariate, respectively. Population was included as a random factor. Two‐way interactions between climatic PCs and sex, as well as sex and body size, were tested. We applied a stepwise backward elimination of non‐significant interactions (*p* > 0.05).

As an additional analysis, to test whether the relationship between environmental variables and the lightness of animals was influenced by shared ancestry among populations, we performed a generalised linear mixed model (glmm) using Markov chain Monte Carlo (R package mcmcglmm v. 2.36; Hadfield 2010, Stone et al. 2011). Unlike in previous analyses, population level averages of lightness values were used as the response variable here. The genetic data of *P. lusitanicus* was obtained from Rato et al. (2025) (estimated for the same animals from the same populations as in this study) and were decomposed to an appropriate genetic distance matrix using the svd function in R, which was included in the analysis as a random factor to account for relatedness among populations (Stone et al. 2011). We used 20,000 iterations and burn‐in (8000) in the estimation of parameters and assumed a flat/non‐informative prior. The spatial autocorrelation, potentially influencing covariation among predictors and responses, was tested separately by Global Moran's I analysis. All the statistical analyses were done using R v.4.4.0 (R Core Team. R Foundation for Statistical Computing 2024).

## 3 Results

### 3.1 Variability in Phenotypic Traits and Environmental Variables Among Populations

Using two separate general linear models with lightness or body size as response variables, and population and sex as predictors, we found significant variation in lightness (*F* = 41.60, df = 20, *p* < 0.001) and body size (*F* = 4.07, df = 20, *p* < 0.001) among different populations sampled (Table S1; Figure S2). The overall mean lightness and body size of all the individuals (*N* = 463) were 10.86 (SD = 1.48) and 51.51 mm (SD = 6.59), respectively. Moreover, females (*N* = 201) tend to be paler (mean ± SD: 11.1 ± 1.49) and smaller in size (mean ± SD: 49.9 ± 6.17 mm) compared to males (*N* = 262, mean lightness ± SD: 10.7 ± 1.45, mean body size ± SD: 52.7 ± 6.65 mm), with variation observed within sex and depending on the population.

We observed considerable environmental variability across the sampled populations (Table S4). Elevation spanned from lowland areas (7 m) to high altitudes (1606 m) while mean ambient temperature had notable seasonal and spatial differences ranging from 7.95°C to 15.65°C (Table S4). Populations also exhibited large variation in hydric environment, with the potential for both relatively dry and wet environments, as well as distinct seasonal wet and dry periods, as indicated by the broad range of annual precipitation (from 773.7 mm to 2299.9 mm) and seasonality patterns (Table S4). Humidity levels also varied widely across the populations (ranging from 0.75 to 2.63), highlighting differences in moisture availability (Table S4). Such diversity in precipitation and humidity is likely a good proxy of local water availability, shaping the local ecosystems and affecting species distribution within the region. A separate analysis of Global Moran's I revealed that spatial autocorrelation among our different populations is very weak (Moran's I _(−0.07,−0.01)_ = −0.04, *p* < 0.05). Thus, we did not account for spatial autocorrelation in the further analyses.

### 3.2 Effect of Environmental Variables on Lightness of Lizards

**FIGURE 3:**
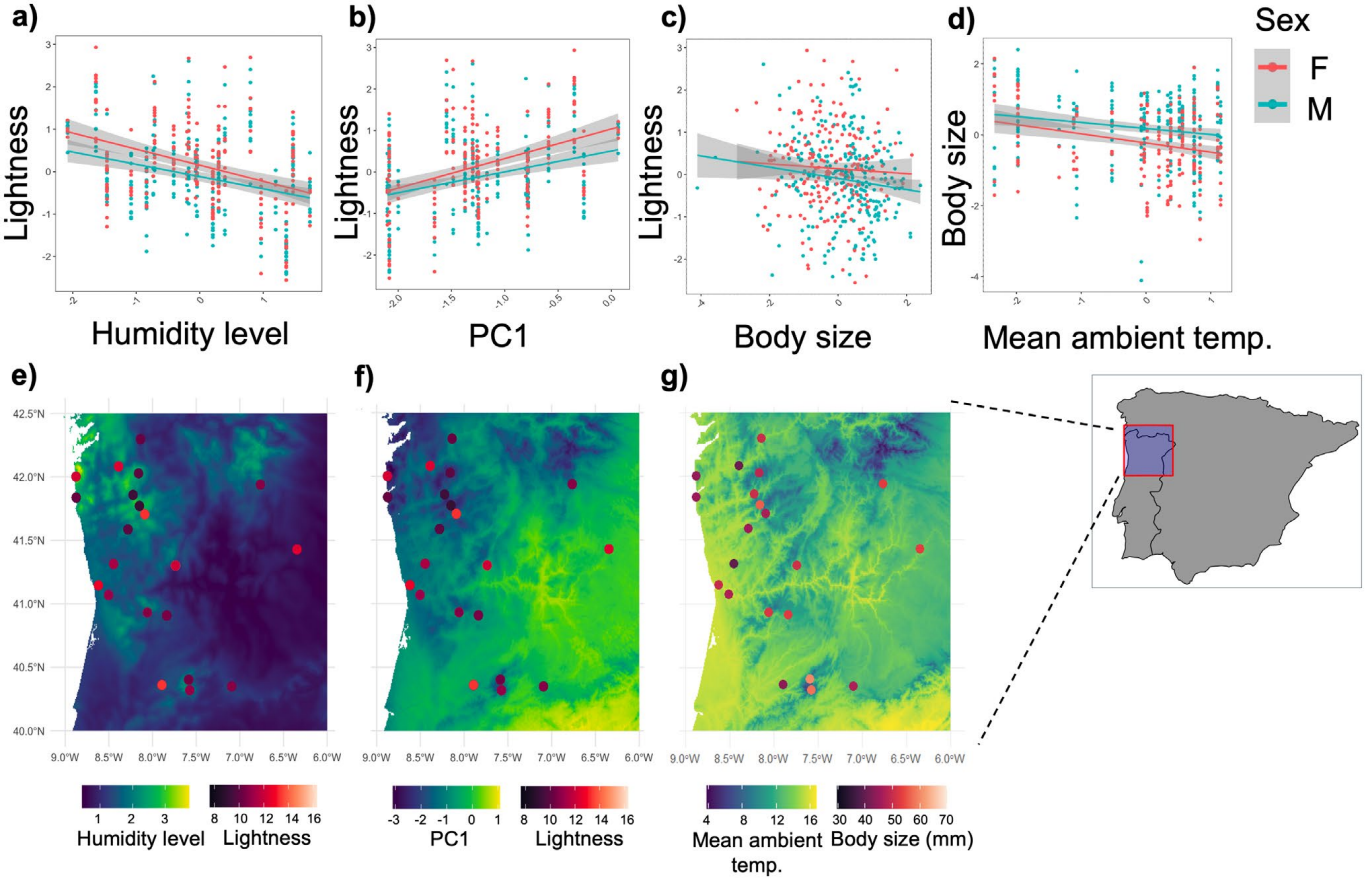
Upper panel: Scatterplots (based on raw data) showing the significant relationships between the (a) lightness of *Podarcis lusitanicus* lizards (*N* = 463) and humidity level of the population locations (*N* = 21), (b) lightness of *P. lusitanicus* and PC1, (c) lightness of *P. lusitanicus* and their body size, and (d) body size of *P. lusitanicus* and mean ambient temperature. The scatterplots include fitted trend lines, separately for males (M) and females (F), and the shaded areas represent the 95% confidence intervals. The continuous variables are standardised by centering and scaling. Lightness and body size were log10 transformed prior to standardisation. Lower panel: (e) relationship between lightness of *P. lusitanicus* lizards and humidity level across the sampled populations, (f) relationship between lightness of *P. lusitanicus* lizards and PC1 across the sampled populations, and (g) relationship between body size of *P. lusitanicus* lizards and mean ambient temperature across the sampled populations. Inset map of Iberian Peninsula at the right end of bottom panel shows the study area within the red coloured rectangle.

We evaluated the different paths connecting environmental variables and the lightness of animals through piecewise structural equation modelling (pSEM; Table 1; Table S2). The structure and direction of paths in the best fit pSEM model (Fisher's C = 9.47, df = 16, *p* = 0.893; Figure 2b) supported Gloger's hypothesis for the humidity component, showing a significant decrease in the lightness of lizards (Figure 3) with an increase in humidity level (standardised SEM coefficient ± 95% CI: *β* = −0.38 ± 0.16, df = 18.35, *p* < 0.05). The same analysis rejected the thermal melanism and photoprotection hypotheses, showing no significant direct effects from either mean ambient temperature (*β* = −0.03 ± 0.34, df = 18.35, *p* = 0.861) or solar radiation (*β* = 0.05 ± 0.45, df = 17.11, *p* = 0.835). The best fit model also supported Bergmann's hypothesis by showing a significant decrease in body size (Figure 3) with an increase in mean ambient temperature (*β* = −0.19 ± 0.08, df = 18.67, *p* < 0.05), and at the same time, lightness decreased with an increase in the body size of animals (*β* = −0.13 ± 0.03, df = 443.38, *p* < 0.001, Figure 3). Thus, mean ambient temperature posed an indirect positive effect on lightness mediated through body size (i.e., higher temperature lowers body size leading to higher lightness, Figure 2b).

The same analysis revealed that humidity levels significantly decreased with increasing solar radiation (*β* = −0.67 ± 0.27, df = 18, *p* < 0.05). The effect of solar radiation translates to an indirect positive effect on lightness through humidity level (Figure 2b). Mean ambient temperature increased significantly with the increase in solar radiation (*β* = 0.20 ± 0.04, df = 17, *p* < 0.001) and decreased with the increase in elevation (*β* = −1.12 ± 0.04, df = 17, *p* < 0.001). In other words, solar radiation posed an indirect negative effect on body size, whereas elevation had an indirect positive effect, both mediated by mean ambient temperature (Figure 2b). Solar radiation significantly increased with the increase in elevation (*β* = 0.57 ± 0.19, df = 19, *p* < 0.01).

Given the final model structure (Figure 2b; Table S2) of the best fit model, sex had a significant effect on lightness and body size (Table 1). Post hoc analysis using pair wise comparison of estimated marginal means revealed that females were lighter (mean ± S.E = 0.20 ± 0.06, df = 441, *p* < 0.01) and attained smaller body size compared to males (mean ± S.E = −0.42 ± 0.09, df = 451, *p* < 0.001). Since sex was a significant factor for both lightness and body size, we also conducted piecewise SEM analyses separately within each sex. The primary findings from these analyses (Figure S3) were qualitatively consistent with the results obtained when both sexes were analysed together. Habitat lightness and vegetation cover were removed from the models since they were not significant in any of the paths.

### 3.3 Principal Components of Environmental Variation

Results from separate mixed modelling with animal lightness as the response variable and principal components representing the major axes of environmental variation as predictors also supported Gloger's hypothesis. The PCA identified two major axes of environmental variation (PC1 and PC2), which together explained 78% of the total variance (PC1: 55%, PC2: 23%, standardised loadings in Table S3). Overall, the positive values of PC1 explained hot areas with warm summers, strong solar radiation, and moderate vegetation cover, but moderate seasonality in temperature and precipitation, while the negative values represented damp and humid areas with wet summers and high annual precipitation (Table S3; Figure S4). Positive values of PC2 represented areas with high temperature seasonality and a moderate range of changes in annual temperature, while negative values of PC2 represented moderately warm areas with low annual precipitation and precipitation seasonality. PC2 also clearly distinguished between high and low altitude areas (Table S3; Figure S4). Lightness varied positively with PC1 (*β* = 0.68 ± 0.27, df = 21.67, *p* < 0.05); increasing in hot areas with warm summer, strong solar radiation, and moderate vegetation cover and decreasing in damp, humid areas with wet summer and high annual precipitation (Figure 3). The lightness of the animals decreased significantly with an increase in body size (*β* = −0.13 ± 0.03, df = 444.54, *p* < 0.001). Body size was not affected by neither of the PCs (see Table S5 for full results). The overall results from these mixed models are consistent with the primary findings from pSEM regarding the variation in lightness.

### 3.4 Accounting for Shared Ancestry Among Populations

Finally, the third analysis, using a generalized linear mixed model (GLMM) and Markov Chain Monte Carlo methods, examined the relationship between environmental factors and animal lightness, accounting for shared ancestry among populations. The results (Table S6) confirmed the negative association of lizard lightness with humidity level and the positive association with the first principal component (PC1) remained significant (*p* < 0.05) with lightness decreasing in humid areas with wet summers and high precipitation, even after controlling for ancestry and gene flow.

## 4 Discussion

The lightness of Lusitanian wall lizards is partially consistent with Gloger's hypothesis (Delhey 2017; Rensch 1938), identifying humidity as a major factor shaping the lightness of this species, while is inconsistent with the predictions for thermal melanism (Clusella‐Trullas et al. 2007, 2009) or photoprotection hypotheses (Law et al. 2020). Following Bergmann's hypothesis, temperature presents an indirect positive effect on lightness mediated through body size. Solar radiation presents an indirect positive effect on lightness mediated through humidity level.

As a recap (Table 2), we addressed whether the lightness of *P. lusitanicus* lizards followed the predictions of ecogeographic hypotheses; namely, whether they are darker in warm and humid areas (Gloger's hypothesis), darker in colder environments (thermal melanism hypothesis), or darker in areas with intense solar radiation (photoprotection hypothesis). We also tested if darker animals are bigger and inhabit in colder areas (Bergmann's hypothesis). While humidity level and body size emerged to be directly linked with lightness (Figures 2b and 3), other environmental factors, such as temperature, elevation, solar radiation and vegetation cover did not exhibit any direct effects. Indirectly, temperature appears to influence this relationship by moderating the negative effect of body size on lightness, while solar radiation regulates the negative effect of humidity level on the lightness of animals (Figures 2b and 3). Also, we detected very weak spatial autocorrelation in our data and the lightness of animals decreased with humidity even after accounting for shared ancestry among populations. Hence, geographic variation in the lightness of lizards across the species' range seems to be driven by differences in environmental pressures acting locally.

**TABLE 2:** Summary of the ecogeographic hypotheses tested to examine the relationship between the lightness of *Podarcis lusitanicus* lizards (*N* = 463) and environmental variables across the sampled populations (*N* = 21). Significant variables directly supporting each hypothesis are indicated by a green tick mark (✓), while non‐significant variables are marked with a red cross (✗). Cells left blank indicate that the variable is not directly associated with the respective ecogeographic hypothesis. Our study simultaneously evaluated predictions from Gloger's hypothesis (darker coloration in warm, humid environments), thermal melanism (darker coloration in colder environments), photoprotection (darker coloration in areas with higher solar radiation intensity), and Bergmann's hypothesis (larger body size in colder environments).

	Temperature	Humidity	Solar radiation	Vegetation index	Habitat lightness
Gloger's	✗	✓		✗	✗
Thermal melanism	✗				
Photoprotection			✗		
Bergmann's	✓				

The simple version of Gloger's hypothesis, originally proposed for endotherms, states that animals are predicted to be darker in warmer and more humid areas, likely expressing increased deposition of melanin pigments (Delhey 2019; Rensch 1938). However, most studies to date support only the effects of humidity (darker animals inhabiting more humid areas) and any other correlated variables representing humidity (e.g., vegetation, humidity, solar radiation), suggesting that the version of Gloger's hypothesis as originally defined (i.e., including temperature as a factor) may not be valid (Delhey 2019). In our study, we attempted to test predictions of the hypothesis based on climatic and other correlated environmental variables, such as vegetation, altitude, solar radiation, etc. We found lizards with darker dorsal colouration occurring more often in more humid areas, characterised by higher humidity level (higher mean annual precipitation and lower levels of mean annual evapotranspiration). Therefore, our findings support the re‐formulated version of Gloger's hypothesis by Delhey et al. (2019), which focuses on humidity as the primary underlying factor, rather than considering both humidity and temperature together. Nevertheless, temperature had an indirect impact on animal lightness, by altering body size, suggesting that higher ambient temperatures possibly result in lighter coloration, by driving decrease in body size, rather than through the direct correlation between temperature and lightness as proposed by Gloger's hypothesis.

The mechanism behind the observed pattern (darker colours in humid areas) could be associated with increased background matching and crypsis, particularly relevant for a species such as *P. lusitanicus* , which is preyed upon by various visual predators (Carretero et al. 2022). Humid areas can be more vegetated with darker backgrounds, leading to higher survival rates for darker individuals in wetter, more vegetated environments through better background matching (Delhey 2018; Friedman and Remeš 2017; McNaught and Owens 2002). However, our results did not support these explanations since neither vegetation cover nor general habitat lightness from the capture sites predicted the lightness of lizards. Nevertheless, overall vegetation cover estimated from remote sensing data might not depict the accurate vegetation structure, although it represents a reasonable compromise (Estrada‐Peña et al. 2014). In this study, we did not assess the habitat lightness of the visual backgrounds for individual lizards due to their considerable movement across diverse backgrounds within their home ranges (Sillero et al. 2020, 2021). Therefore, the lightness of the most commonly used backgrounds, such as rocks or granite walls, could serve as an indicator of the overall lightness of their visual background. Alternatively, increased melanisation in animals may provide a protective barrier against microbial infections (Goldenberg et al. 2024; Mackintosh 2001; Nappi and Christensen 2005), particularly in humid environments where the prevalence of pathogens is high (Horrocks et al. 2015), though further screening would be required to confirm this. In fact, melanism is often functionally and genetically linked to other characteristics, such as stress resilience, anti‐pyretic, anti‐inflammatory and antioxidant responses, hormone profiles and energy regulation (Ducrest et al. 2008; Raia et al. 2010; Wittkopp and Beldade 2009). These pleiotropic effects can result in darker animals having enhanced physiological traits, including stronger immune defences. Therefore, in environments where humidity drives pathogen fluctuations, natural selection may favour darker individuals with these advantages. Recently, Rato et al. (2025) reported no evidence of selection in the MC1R gene, which is involved in melanin production, within the same *P. lusitanicus* populations. This suggests that further research is needed to determine whether selection favours any pleiotropic effects of melanin in humid environments, with melanisation potentially being a by‐product of other adaptive traits (Ducrest et al. 2008).

The association between lizard lightness and the environment can also be modulated by body size (Goldenberg et al. 2022). Since both lightness and body size are major factors in thermoregulation, the relationship between melanisation, body size and ambient temperature may play a significant role in shaping the biogeographic patterns of lightness variation (Clusella‐Trullas et al. 2007). Although we did not find direct support for the thermal melanism hypothesis, our results provided indirect evidence by aligning with Bergmann's hypothesis, which suggested that lizards were larger in colder areas, and the larger individuals were also darker (Figure 3). Larger animals, with their greater thermal inertia, are more effective at retaining heat, whereas smaller ectotherms are better at rapidly gaining heat (Zamora‐Camacho et al. 2014; Carothers et al. 1997). Larger animals require more time to reach optimal body temperatures; being darker can compensate for this lag in heat uptake, as darker animals heat up more quickly (González‐Morales et al. 2021). Ambient temperature may influence the optimal balance between body size and colouration, with body size potentially mediating how lizards adapt their lightness to different thermal environments. This mechanism, potentially explaining the pattern observed by Bergmann and some researchers after him (Ashton and Feldman 2003; Bergmann 1847; Goldenberg et al. 2022), might also explain why larger lizards are darker in colder areas. Rapid heat uptake enhances the fitness of ectotherms, for example, improving the ability to defend territory, find mates, feed and evade predators (Clusella‐Trullas et al. 2007). Although melanism appears to provide a greater thermal advantage for larger ectotherms, the increased thermal inertia in bigger animals reduces the need for rapid heat absorption over time, limiting the overall advantages of melanisation (Clusella‐Trullas et al. 2007, 2008). Also, non‐adaptive explanations on body size based on life history have been suggested for lizards (Angilletta et al. 2004). Overall, future studies assessing heating and cooling rates in *P. lusitanicus* lizards are required to confirm the role of ambient temperature in the evolution of lightness and body size. Moreover, Goldenberg et al. (2024) observed that in cordylid lizards inhabiting high‐specific heat capacity substrates (colder environments), larger individuals exhibit darker ventral coloration than smaller ones. These authors found dorsally lighter individuals close to the equator in warmer environments (Goldenberg et al. 2024). Further studies are needed to investigate the role of substrate heat capacities and ventral reflectance of animals in greater detail.

In habitats with high UV exposure, melanisation has been suggested to serve a photoprotective role against harmful solar radiation (Law et al. 2020; Wang et al. 2008). However, our study failed to provide any evidence specifically supporting photoprotection, as no significant direct effect from solar radiation on lightness was recovered. Interestingly, solar radiation had a positive indirect effect on lightness, mediated through humidity level; higher solar radiation lowers humidity level leading to higher lightness, suggesting a more complex role for solar radiation than simple photoprotection. Given this, the primary function of melanisation in our lizard population may lean more towards thermoregulation rather than UV protection. This could mean that in areas with both high solar radiation and lower humidity, lizards might not need to be as dark as they would in environments with lower solar exposure and higher humidity. Approximately 55% of radiant energy from direct sunlight falls within the near‐infrared (NIR) spectrum (700–2500 nm), while the remaining 45% lies within the animal‐visible range (300–700 nm, Stuart‐Fox et al. 2017). To fully understand the thermal consequences of lightness variation, it is essential to consider how an animal's surface interacts with the entire solar spectrum, not just the visible wavelengths. Darker animals, which typically absorb more NIR radiation, may benefit from faster heat uptake (Goldenberg et al. 2021; Shawkey et al. 2017). This may support thermoregulation under environments with lower solar exposure and higher humidity. Although technical constraints limited our ability to obtain NIR data, further research should explore the intricate relationships between UV–Vis and NIR reflectance, environmental factors such as humidity and solar radiation, and thermoregulation to better understand how these variables shape melanisation patterns.

Our study contributes valuable insights into the ecogeographic factors shaping the lightness variation in *P. lusitanicus* lizards. Darker colouration of *P. lusitanicus* lizards can represent an adaptative response to humid areas. The finding that lizards are darker in high‐humidity environments, with temperature having no direct effect but influencing coloration indirectly through body size, highlights a key contradiction between Gloger's rule and the thermal melanism hypotheses. Animals are larger in colder environments and larger animals are darker as well. The interdependence of phenotypes with shared ancestry and continuous gene flow among populations can blur the signal of local selection in phenotypic variation (Stone et al. 2011). Here, phylogeographic evidence recovers high gene flow between *P. lusitanicus* populations, while also revealing some phylogeographic patterns related to microrefugia from the late Pleistocene (Rato et al. 2025). However, after statistically controlling for these phylogenetic effects, the signal of the local environmental conditions in lightness remained. Ecogeographic hypotheses, which describe broad geographical patterns affecting phenotypic traits, can sometimes obscure important underlying microenvironmental trends, as well as the mechanistic and functional explanations behind these patterns (Goldenberg et al. 2022). For instance, multiple interpretations and lack of evidence for a clear adaptive mechanism makes the general applicability of Gloger's hypothesis more difficult. So, we advise caution while applying ecogeographic hypotheses to interpret biogeographic trends in the variation of phenotypic traits.

## 5 Conclusions

Overall, our results provide quantitative evidence supporting the role of environmental variables in shaping the lightness in *P. lusitanicus* lizards. We have found partial support for Gloger's hypothesis, particularly the role of humidity in influencing lightness of lizards. Conversely, we found no direct evidence supporting the thermal melanism hypothesis, as there was no direct correlation between temperature and lightness. However, our results offered indirect support by aligning with Bergmann's hypothesis, suggesting that larger lizards are found in colder regions, and larger individuals also tend to be darker, possibly responding to the thermal environment. Finally, we found no support for the photoprotection hypothesis, as solar radiation did not have a direct impact on lightness, though it had an indirect positive effect on lightness, modulated through the effects of humidity. Given that darker animals may benefit more from global warming through increased activity than their lighter counterparts (Mader et al. 2022), in areas where cold temperatures are currently limiting their activity, it is essential to investigate the complex interactions between lightness, body size, and environmental variables. Future research should explore the physiological, genetic, and life‐history factors that influence these interactions, as well as the evolutionary forces like gene flow and genetic drift that complicate them. Indeed, a more integrated approach will provide deeper insights into the adaptive significance of melanism in lizards across diverse environments, beyond simple correlations suggested by ecogeographic hypotheses.

## Author Contributions

**Lekshmi B. Sreelatha:** conceptualization (equal), data curation (lead), formal analysis (lead), investigation (equal), writing – original draft (lead), writing – review and editing (equal). **Pedro Tarroso:** formal analysis (supporting), investigation (equal), writing – review and editing (equal). **Ossi Nokelainen:** conceptualization (equal), formal analysis (supporting), investigation (equal), writing – review and editing (equal). **Zbyszek Boratyński:** conceptualization (equal), data curation (supporting), formal analysis (supporting), investigation (equal), writing – review and editing (equal). **Miguel Angel Carretero:** conceptualization (equal), data curation (supporting), formal analysis (supporting), funding acquisition (lead), investigation (equal), writing – review and editing (equal).

## Ethics Statement

All applicable international, national and/or institutional guidelines for the care and use of animals were followed. Collecting permits 536 / 2022 / CAPT and EB‐042/2023 were provided by the Institute for Nature Conservation and Forests (ICNF, Portugal) and the General Council of Galicia (Spain), respectively. This study was approved by the ethical guidelines of the University of Porto.

## Conflicts of Interest

The authors declare no conflicts of interest.

## Supporting information


Data S1.

## Data Availability

The data that support the findings of this study are openly available in “figshare” at https://figshare.com/s/3e3d57d2d2d3253a6309.
