# Correction to “Environmental Gradients in Lizard Colouration”

**DOI:** 10.1002/ece3.71437

**Published:** 2025-05-12

Sreelatha, L.B., Tarroso, P., Nokelainen, O., Boratyński, Z. and Carretero, M.A. (2025), Environmental Gradients in Lizard Colouration. *Ecol Evol*, 15: e71012. https://doi.org/10.1002/ece3.71012

In the fifth paragraph of the Discussion section, there is a typo in one of the citations. The year “2024” in Goldenberg et al. (2024) should be changed to “2023.” Additionally, the full reference for Goldenberg et al. (2023) needs to be included in the list of references. The corrected text is shown below.

Moreover, Goldenberg et al. (2023) observed that in cordylid lizards inhabiting high‐specific heat capacity substrates (colder environments), larger individuals exhibit darker ventral coloration than smaller ones. These authors found dorsally lighter individuals close to the equator in warmer environments (Goldenberg et al. 2023).

We apologize for this error.
