# Supplementary material for: Environmental Gradients in Lizard Colouration
Source: Ecol Evol. 2025 Feb 28;15(3):e71012. doi: 10.1002/ece3.71012 (PMC11871090; doi:10.1002/ece3.71012)
Supplement: Supplementary file 1 — Data S1. [file ECE3-15-e71012-s001.docx]

**Supporting information**

**Table S1.** Mean lightness and mean body size of *Podarcis lusitanicus* lizards (N= 463) captured from 21 natural populations across the north-western Iberian Peninsula.

| SL. No. | Population | Number of animals | | Lightness (mean ± s.d) | | Body size (mm, mean ± s.d)) | |
| --- | --- | --- | --- | --- | --- | --- | --- |
|  |  | Male | Female | Male | Female | Male | Female |
| 1 | Barrocal do Douro | 6 | 4 | 12.01 ± 0.56 | 12.03 ± 0.31 | 50.81 ± 3.86 | 52.53 ± 5.30 |
| 2 | Castro Laboreiro | 5 | 2 | 9.64 ± 0.83 | 9.83 ± 0.14 | 54.93 ± 4.21 | 46.25 ± 3.72 |
| 3 | Crestuma | 13 | 8 | 10.88 ± 0.56 | 11.89 ± 0.65 | 50.18 ± 5.04 | 46.01 ± 5.10 |
| 4 | Fafiao | 11 | 7 | 12.87 ± 1.19 | 12.86 ± 1.46 | 52.21 ± 2.64 | 47.58 ± 3.10 |
| 5 | Leonte | 9 | 5 | 10.25 ± 0.74 | 9.78 ± 0.54 | 50.06 ± 9.51 | 51.61 ± 5.27 |
| 6 | Lindoso/Poco da Gola | 6 | 11 | 8.96 ± 0.59 | 9.62 ± 0.90 | 58.60 ± 0.80 | 47.14 ± 8.33 |
| 7 | Miradouro dos Piornos | 12 | 9 | 11.23 ± 1.85 | 12.12 ± 1.60 | 55.94 ± 10.10 | 57.94 ± 9.56 |
| 8 | Moledo | 19 | 13 | 8.46 ± 0.46 | 8.98 ± 0.77 | 50.28 ± 5.96 | 48.55 ± 4.28 |
| 9 | Montesinho | 6 | 6 | 10.29 ± 1.28 | 11.04 ± 0.97 | 53.82 ± 3.00 | 49.96 ± 4.08 |
| 10 | Oia | 20 | 16 | 11.40 ± 0.74 | 11.34 ± 0.87 | 52.67 ± 7.68 | 52.81 ± 6.02 |
| 11 | Oliveira do Hospital | 8 | 11 | 11.78 ± 1.38 | 12.20 ± 1.12 | 54.97 ± 4.89 | 49.44 ± 5.67 |
| 12 | Parada de Ester | 9 | 1 | 10.97 ± 1.47 | 10.23 | 54.71 ± 3.03 | 52.49 |
| 13 | Parque das Virtudes, Porto | 14 | 10 | 10.21 ± 0.92 | 10.73 ± 0.71 | 55.75 ± 6.37 | 52.38 ± 5.56 |
| 14 | Pendilhe | 18 | 7 | 10.08 ± 0.90 | 10.23 ± 0.64 | 52.31 ± 6.07 | 50.35 ± 4.28 |
| 15 | Povoa de Lanhoso | 20 | 19 | 9.62 ± 0.59 | 10.38 ± 0.77 | 52.33 ± 5.96 | 48.51 ± 5.46 |
| 16 | Ribadavia | 17 | 17 | 10.31 ± 0.85 | 10.42 ± 1.06 | 53.87 ± 2.42 | 51.35 ± 3.14 |
| 17 | Sabugal | 14 | 7 | 10.23 ± 0.83 | 10.56 ± 0.83 | 50.23 ± 9.68 | 49.27 ± 4.99 |
| 18 | Santo Tirso | 8 | 8 | 10.94 ± 0.53 | 11.08 ± 0.52 | 53.57 ± 4.95 | 43.44 ± 3.72 |
| 19 | Setados | 14 | 11 | 11.84 ± 1.66 | 12.49 ± 1.17 | 45.28 ± 4.87 | 45.14 ± 5.95 |
| 20 | Vale do Rossim | 22 | 18 | 10.94 ± 0.78 | 11.13 ± 1.06 | 56.57 ± 7.29 | 53.74 ± 6.19 |
| 21 | Vila Real | 11 | 11 | 12.87 ± 1.08 | 13.79 ± 1.14 | 52.74 ± 5.68 | 48.28 ± 4.14 |

**Table S2**. Model list of the piecewise SEM showing the paths between lightness of *Podarcis lusitanicus* lizards (N = 463) and environmental variables across the sampled populations (N = 21). We indicate the modelling method, response variables, predictors and random factor used in each model. Variables in bold were added to the models based on the tests of d-separation. We also provide marginal (R^2^m) and conditional (R^2^c) R^2^ for each model. The initial model had a Fisher’s C statistic of = 220.74 with *p* < 0.001 and 34 degrees of freedom with an AIC of 2449.94. Since the initial model did not fit the data structure (significant *p* value), we added the missing paths between variables based on Shipley’s test for d-separation. Once the model converged (Fisher's C = 22.124, df = 26, *p* = 0.682), we discarded the non-significant predictors through stepwise backward elimination. Habitat brightness and vegetation index were removed from the models since they were not significant in any of the paths. The final model had a Fisher’s C statistic of = 9.47 with *p* = 0.893 and 16 degrees of freedom and an AIC of 2299.04. All continuous predictors were standardised (centered and scaled) prior to the analysis. Lightness and body size were log10 transformed prior to standardisation and fitted into linear mixed models (LMM) with population as random factor using the package lme4 (Bates et al., 2015). All the other relations were fitted into general linear models (GLM) using the built in glm() function in R. Sex is included as a categorical factor. We specified correlated error between mean ambient temperature and elevation, to account for the unmeasured sources of variance between these two variables.

| Method | Response | Predictors | Random | R^2^m | R^2^c |
| --- | --- | --- | --- | --- | --- |
| LMM | Lightness | Humidity level, mean ambient temperature, body size, sex | Population | 0.16 | 0.7 |
| LMM | Body size | Mean ambient temperature, sex | Population | 0.08 | 0.2 |
| GLM | Humidity level | Elevation, **solar radiation** | - | 0.26 | - |
| GLM | Solar radiation | Elevation | - | 0.31 | - |
| GLM | Mean ambient temperature | Elevation, solar radiation, **humidity level** | - | 0.99 | - |

**Table S3:** Results from principal component analysis (PCA) on the environmental variables to identify the major axes of environmental variation across the populations of *Podarcis lusitanicus*. The first two principal components (PC1 and PC2, each explaining > 5% of the variance in the data and have eigenvalues >1) explained 78% of variance (PC1: 55%, PC2: 23%) observed.

| Ecogeographic variables | PC1 | PC2 |
| --- | --- | --- |
| Mean ambient temperature | 0.73 | -0.60 |
| Temperature Seasonality | 0.63 | 0.70 |
| Max Temperature of Warmest Month | 0.95 | -0.08 |
| Temperature Annual Range | 0.70 | 0.62 |
| Annual Precipitation | -0.82 | -0.33 |
| Precipitation Seasonality | 0.47 | -0.66 |
| Precipitation of Warmest Quarter | -0.91 | 0.12 |
| Vegetation cover | 0.63 | -0.09 |
| Solar radiation | 0.84 | -0.11 |
| Humidity level | -0.90 | -0.26 |
| Elevation | -0.35 | 0.81 |

**Table S4.** Environmental variables representing the 21 natural populations of *Podarcis lusitanicus* sampled across the north-western Iberian Peninsula (Temp. = Temperature, Precip. = Precipitation).

| Sl.No | Population | Latitude | Longitude | Elevation | Vegetati-on index | Habitat lightness | Solar radiation | Humidity level | Mean Ambient temp. | Temp. seasona- lity | Annual precip. | Precip. seasonality | |
| --- | --- | --- | --- | --- | --- | --- | --- | --- | --- | --- | --- | --- | --- |
| 1 | Barrocal do Douro | 41.4286 | -6.3457 | 685 | 0.08 | 14.50 | 184793 | 0.75 | 13.05 | 587.30 | 773.70 | 46.80 |  |
| 2 | Castro Laboreiro | 42.0276 | -8.1598 | 946 | -0.02 | 11.46 | 169316 | 2.37 | 10.15 | 457.80 | 2061.60 | 51.60 |  |
| 3 | Crestuma | 41.0685 | -8.5034 | 14 | -0.03 | 10.78 | 178431 | 1.57 | 15.65 | 407.10 | 1449.30 | 51.40 |  |
| 4 | Fafiao | 41.7053 | -8.0892 | 526 | -0.02 | 17.10 | 172104 | 2.17 | 13.05 | 467.80 | 2037.80 | 54.20 |  |
| 5 | Leonte | 41.7713 | -8.15 | 940 | -0.03 | 12.00 | 172501 | 2.63 | 10.75 | 457.30 | 2299.90 | 54.00 |  |
| 6 | Lindoso/Poco da Gola | 41.8578 | -8.2228 | 369 | -0.02 | 13.35 | 169819 | 2.25 | 13.15 | 460.90 | 2149.90 | 53.00 |  |
| 7 | Miradouro dos Piornos | 40.319 | -7.5733 | 1606 | -0.05 | 12.44 | 190682 | 1.97 | 7.95 | 511.00 | 1659.10 | 59.00 |  |
| 8 | Moledo | 41.837 | -8.8739 | 7 | 0.00 | 11.96 | 171565 | 2.45 | 14.75 | 292.80 | 1681.40 | 50.10 |  |
| 9 | Montesinho | 41.939 | -6.7648 | 1011 | -0.03 | 19.18 | 179028 | 1.68 | 10.65 | 546.60 | 1526.10 | 52.80 |  |
| 10 | Oia | 42.0024 | -8.876 | 18 | 0.03 | 15.80 | 169769 | 2.50 | 14.75 | 277.00 | 1624.30 | 50.60 |  |
| 11 | Oliveira do Hospital | 40.3603 | -7.8925 | 378 | 0.01 | 14.37 | 189927 | 1.43 | 14.25 | 497.40 | 1480.30 | 55.20 |  |
| 12 | Parada de Ester | 40.9317 | -8.0588 | 357 | -0.02 | 12.94 | 181882 | 1.42 | 14.25 | 478.60 | 1436.40 | 53.20 |  |
| 13 | Parque das Virtudes, Porto | 41.1451 | -8.6191 | 50 | 0.00 | 17.74 | 176917 | 1.75 | 15.55 | 380.70 | 1446.10 | 51.50 |  |
| 14 | Pendilhe | 40.908 | -7.837 | 744 | 0.00 | 11.38 | 184643 | 1.36 | 11.95 | 495.30 | 1304.80 | 53.60 |  |
| 15 | Povoa de Lanhoso | 41.5861 | -8.2812 | 362 | -0.01 | 12.80 | 172664 | 1.88 | 13.95 | 457.30 | 1873.40 | 52.60 |  |
| 16 | Ribadavia | 42.2968 | -8.1374 | 74 | -0.01 | 16.05 | 161691 | 1.06 | 14.35 | 438.40 | 1052.50 | 50.50 |  |
| 17 | Sabugal | 40.3496 | -7.0936 | 736 | 0.02 | 12.57 | 194687 | 1.24 | 12.95 | 585.00 | 1266.10 | 55.50 |  |
| 18 | Santo Tirso | 41.3129 | -8.446 | 380 | -0.01 | 9.03 | 175369 | 1.74 | 13.85 | 437.70 | 1600.40 | 50.90 |  |
| 19 | Setados | 42.0825 | -8.388 | 57 | -0.02 | 17.68 | 166209 | 1.69 | 14.95 | 430.80 | 1689.80 | 50.00 |  |
| 20 | Vale do Rossim | 40.4027 | -7.5865 | 1435 | -0.03 | 14.66 | 189890 | 1.92 | 8.75 | 509.90 | 1662.60 | 58.60 |  |
| 21 | Vila Real | 41.3011 | -7.7366 | 405 | 0.00 | 13.34 | 178458 | 0.97 | 13.55 | 515.30 | 979.70 | 53.90 |  |

**Table S5:** Effect of climatic principal components (PC1 and PC2) on the lightness of *Podarcis lusitanicus* lizards (N = 463) from mixed models. Population was included as a random factor. Interaction between sex and PCs as we all as sex and body size were not significant and were discarded from the analysis. Lightness and body length were log10 transformed, centred and scaled. Sex was included as a categorical factor. MS refers to mean square, and F _(n,d )_ to F statistic with degrees of freedom for numerator and denominator

| Dependent variable | Predictors | *M.S* | *F _(n,d)_* | *P* |
| --- | --- | --- | --- | --- |
| Lightness | PC1 | 2.06 | 5.84 _(1, 19.56)_ | 0.026 |
|  | Sex | 4.38 | 12.41 _(1, 441.75)_ | <.001 |
|  | Body size | 6.47 | 18.32 _(1, 444.54)_ | <.001 |
| Body size | PC1 | 0.79 | 0.93 _(1, 21.62)_ | 0.344 |
|  | PC2 | 2.64 | 3.12 _(1, 18.23)_ | 0.094 |
|  | Sex | 19.63 | 23.22 _(1, 450.37)_ | <.001 |

PC1 had a significant effect on the lightness of lizards (F _(1, 19.56)_ = 5.84, p<0.05). Males and females differed in lightness (F _(1, 441.75)_ = 4.38, p<0.001). Within sexes, lightness decreased with decrease in PC1 (Figure 3b) in both males (β= 0.60 ± 0.26, df = 19.76, p<0.05) and females (β= 0.66 ± 0.27, df = 20.28, p<0.05). On the contrary, PC2 had no significant effects on lightness (F _(1, 18.91)_ = 1.53, p = 0.232). Lightness varied along with body size as well (F _(1, 444.54)_ = 18.32, p<0.001); lightness decreased significantly with increase in body size (β= -0.13 ± 0.03, df = 444.54, p<0.001) Body size was not affected by PC1 and PC2, but males and females differed in body size (F _(1, 450.37)_ = 19.63, p<0.001). Pairwise comparison of the estimated marginal means revealed that females have smaller body size compared to males (mean ± S.E = -0.42 ± 0.09, df = 450, p<0.001). We applied stepwise backward elimination method to reduce complexity of the final model. Two-way interactions of sex with PCs, sex with body size and PCs with body size were non-significant and were removed from the model.

**Table S6.** Posterior means, confidence intervals, and p-value for regressions between lightness and humidity level (model number 1), lightness and PC1 (model number 2), lightness and PC2 (model number 3). We used 20000 iterations and burn-in (8000) in the estimation of parameters, as well as a ﬂat/non-informative prior.

| Model number | Predictor | Posterior Mean | 95% CI  (Lower) | 95% CI  (Upper) | Effective  Sample Size | pMCMC |
| --- | --- | --- | --- | --- | --- | --- |
| 1 | Humidity level | -0.38 | 0.01 | 0.72 | 1920 | 0.039 |
|  | Mean ambient temp. | -0.05 | -0.45 | 0.43 | 2111 | 0.814 |
|  | Body size | -0.10 | -1.29 | 0.96 | 2062 | 0.856 |
| 2 | PC1 | 0.66 | 0.10 | 1.32 | 1920 | 0.035 |
|  | Body size | -0.03 | -0.98 | 0.92 | 1959 | 0.957 |
| 3 | PC2 | 0.25 | -0.23 | 0.76 | 1920 | 0.301 |
|  | Body size | -0.24 | -1.35 | 0.79 | 1920 | 0.657 |

**Figure S1:** Sampling locations (N=21) of *Podarcis lusitanicus* across the north-western Iberian Peninsula

**
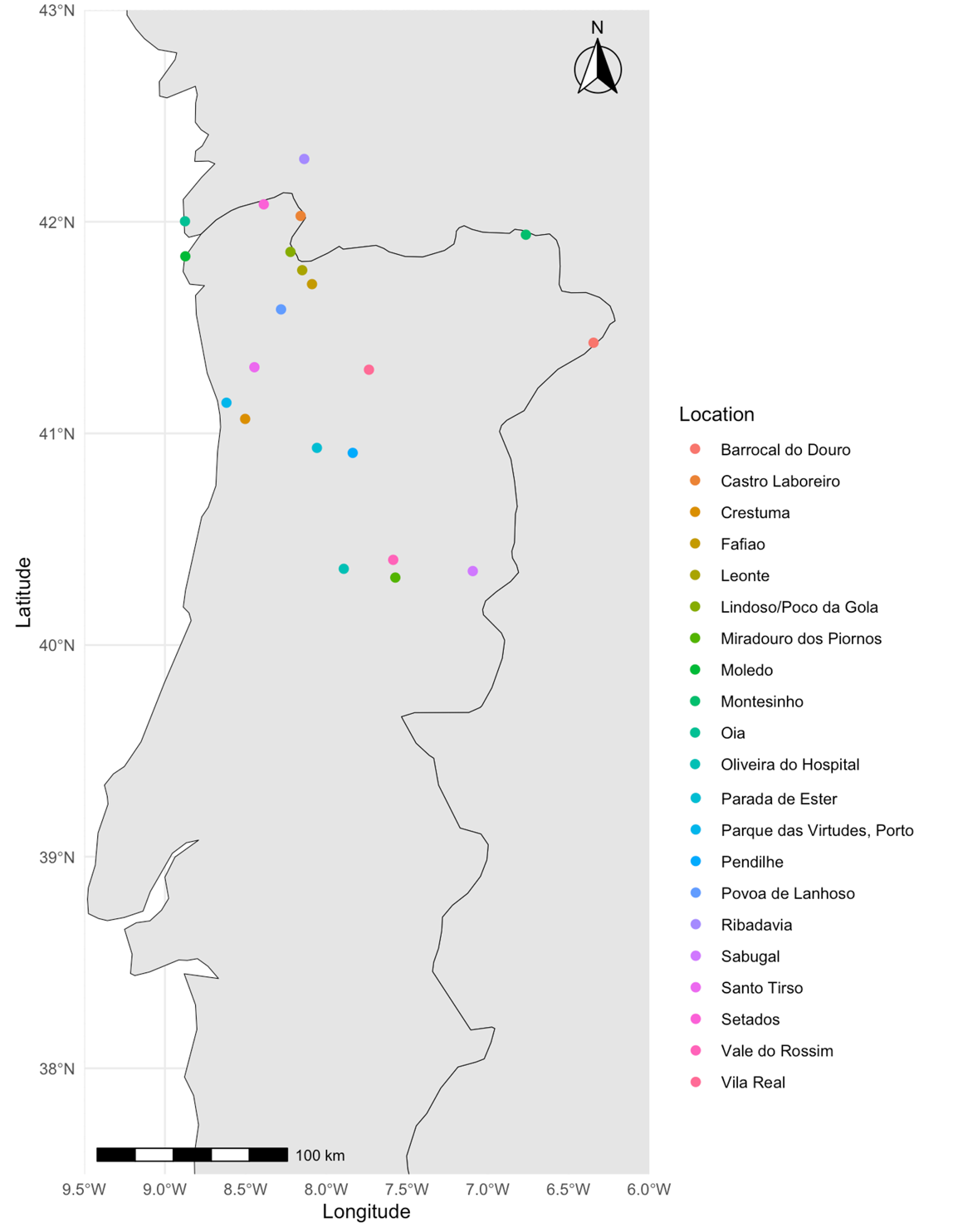
**

**Figure S2:** Variation in reflectances (a) Red channel, R; b) Green channel, G; c) Blue channel, B; d) Ultraviolet red, UVR), e) lightness and f) body size of lizards (N = 463) across 21 natural populations across the north-western Iberian Peninsula


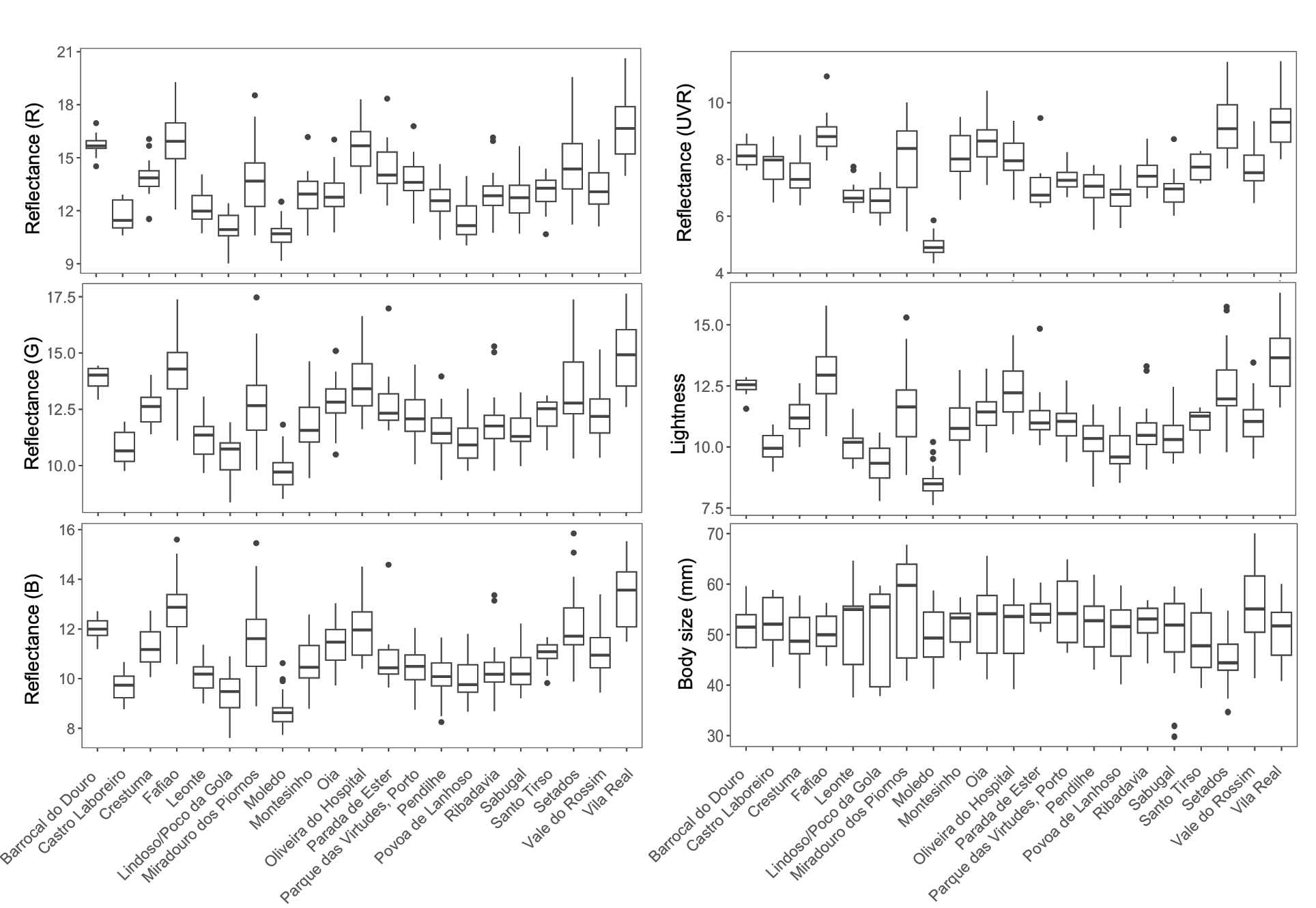


**a)**

**b)**

**c)**

**d)**

**e)**

**f)**

**Figure S3:** Best selected path diagrams from the piecewise SEM (pSEM) model showing the direct and indirect relationships between lightness, body size (measured by snout-vent length) of *Podarcis lusitanicus* lizards, within each sex (males: N = 262, females: N = 201), with environmental variables across the sampled populations (N = 21). a) best fit pSEM model for males (AIC = 1394.43, Fisher's C = 6.75, df = 10, *p* = 0.749), b) best fit pSEM model for females (AIC = 1067.32, Fisher's C = 6.75, df = 10, *p* = 0.749). Each single-headed arrow represents a direct causal path. Continuous lines represent significant paths and arrow thickness is proportional to their effect size. Broken line shows the non-significant path. The numbers near the arrows show standardised path coefficients (with 95% C.I) indicating the magnitude of the effect of one variable upon another. Red and green arrows represent the negative and positive relations, respectively.

**Figure S4:** Summary of climatic variation in the study area. In general, PC1 (a) largely reflected the bioclimatic division in the Iberian Peninsula (Sillero *et al.,* 2009), separating the coastal areas exposed to the Atlantic climate from the continental ones with more Mediterranean characters. PC2 (b) indicated the degree of continentality capturing the gradient from the coastal areas to farther inland.


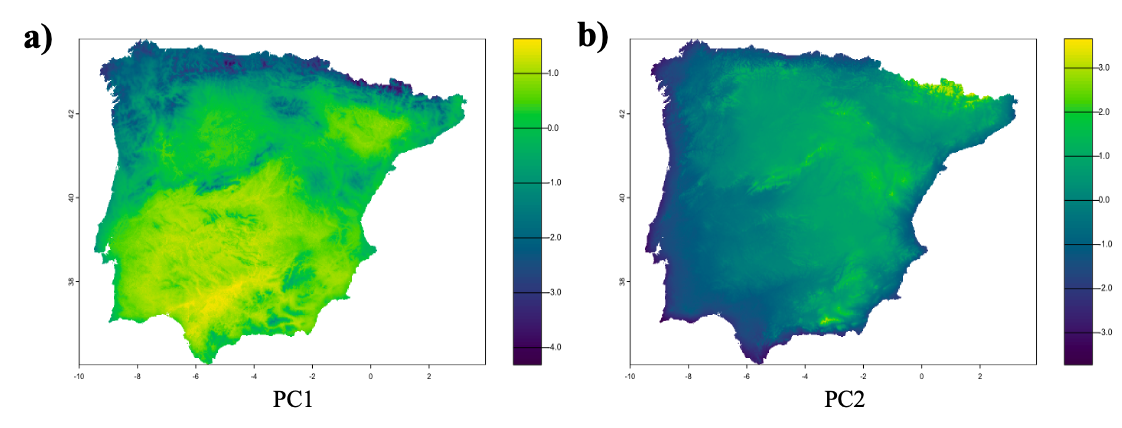


**References**

Bates, D., Mächler, M., Zurich, E., Bolker, B. M., & Walker, S. C. (2015). Fitting Linear Mixed-Effects Models Using lme4. *Journal of Statistical Software*, *67*(1), 1–48. https://doi.org/10.18637/jss.v067.i01

Sillero, N., Brito, J.C., Skidmore, A.K. & Toxopeus, B. (2009). Biogeographical patterns derived from remote sensing variables: the amphibians and reptiles of the Iberian Peninsula. *Amphibia-Reptilia*, 30: 185-206.
